# NOD1 deficiency impairs CD44a/Lck as well as PI3K/Akt pathway

**DOI:** 10.1038/s41598-017-03258-y

**Published:** 2017-06-07

**Authors:** Yi Wei Hu, Xiao Man Wu, Shi Si Ren, Lu Cao, Pin Nie, Ming Xian Chang

**Affiliations:** 1 State Key Laboratory of Freshwater Ecology and Biotechnology, Institute of Hydrobiology, Chinese Academy of Sciences, Wuhan, Hubei Province 430072 China; 2 Graduate University of Chinese Academy of Sciences, Beijing, 100039 China

## Abstract

Pattern recognition receptors (PRRs) are crucial for host defense and tissue homeostasis against infecting pathogens. PRRs are highly conserved cross species, suggesting their key roles in fundamental biological processes. Though much have been learned for NOD1 receptor in the innate and adaptive immune responses, the roles of NOD1 during embryonic and larval stages remain poorly understood. Here, we report that NOD1 is necessary for the modulation of PI3K-Akt pathway and larval survival in zebrafish. Transcriptome analysis revealed that the significantly enriched pathways in *NOD1* ^−/−^ zebrafish larvae were mainly involved in metabolism and immune system processes. Biochemical analysis demonstrated that NOD1 was required for the expression of CD44a that, in turn, activated the PI3K-Akt pathway during larval development. Conversely, over-expression of CD44a in NOD1-deficient zebrafish restored the modulation of the PI3K-Akt pathway and improved larval survival. Collectively, our work indicates that NOD1 plays a previously undetected protective role in larval survival through CD44a-mediated activation of the PI3K-Akt signaling.

## Introduction

NOD-like receptors (NLRs) are a large family of intracellular pattern recognition receptors (PRRs) with a characteristic arrangement of nucleotide-binding domain (NBD) and leucine-rich repeat (LRR) regions. The most prominent function of NLRs is to recognize intracellular pathogen-associated molecular patterns (PAMPs)^1^. Phylogenetic analysis has revealed 3 distinct subfamilies within the NLR family: the NODs (NOD1-2, NOD3/NLRC3, NOD4/NLRC5, NOD5/NLRX1, CIITA), the NLRPs (NLRP1-14, also called NALPs) and the IPAF subfamily consisting of IPAF (NLRC4) and NAIP^2, 3^. Of them, NOD1 is the first characterized NLR member^4^. In mammals, NOD1 senses D-glutamyl-meso-diaminopimelic acid (DAP), a fragment of bacterial peptidoglycan (PGN) found in all Gram-negative bacteria and a few Gram-positive bacteria^5, 6^. Upon ligand binding, NOD1 interacts with receptor-interacting protein 2 (RICK/RIP2) through CARD-CARD domain interaction, which triggers the activation of NF-κB and MAPK (mitogen-associated protein kinase) pathways^7, 8^. These signaling cascades potently up-regulate the production of pro-inflammatory cytokines and antimicrobial molecules, constituting the innate immune response.

Significant advances have been achieved regarding the function of NOD1 signaling pathway, including its role in innate immune responses to bacterial and protozoan parasite infections^9, 10^, in shaping adaptive immune responses towards bacteria and bacterial-derived constituents^11, 12^, and in autophagy and inflammatory signaling in response to invasive bacteria^13, 14^. NOD1 signaling was also reported to be involved in insulin resistance in mammalian adipocytes^15–17^. In contrast, very little is known about the role and mechanisms of NOD1 in early stage of ontogeny, although one recent report found that the activation of NOD1 signaling induced fetal growth restriction and death in mice^18^.

NOD1 genes have been cloned from rainbow trout^19^, goldfish^20^, orange-spotted grouper^21^, olive flounder^22^, channel catfish^23, 24^, rohu^25^ and grass carp^26^. The teleost NOD1 has a structure with signature conserved domains similar to the mammalian counterpart. Experiments entailing loss-of-function or gain-of-function studies indicate that piscine NOD1 is crucial for the expression of pro-inflammatory cytokines^19, 22^ and a shared antibacterial function^22, 27^. In zebrafish, morpholino-mediated depletion of NOD1 does not affect embryonic development and larval survival rate, even in response to bacterial infection. However, the splice-blocking morpholino may not penetrate target cells at late stages of zebrafish larvae^27^. Whether piscine NOD1 contributes to immune defense and relevant mechanism during early embryonic development and larval survival remains to be resolved.

CD44 is a transmembrane adhesion molecule and the major receptor for hyaluronan. In addition to the crucial role of mediating T-cell extravasation^28, 29^, regulating effector T-cell responses^30^ and T-cell development^31^, CD44 also activates the PI3K-Akt pathway which is associated with cell survival in various cell types including leukemia^32^ and T-cell^33^. NLRs and Toll-like receptors (TLRs) have shaped our current understanding of innate regulation of adaptive immunity^34^. Recent studies have identified a cross talk among CD44, TLRs and the PI3K-Akt pathway in pathological conditions^35, 36^. However, whether the functional correlation among NOD1, CD44 and PI3K-Akt pathway exists in the immune system, especially during early ontogenesis, is still unclear.

Our previous report showed the high expression of NOD1 in the embryonic and larval stage of zebrafish^37^. This promoted us to generate *NOD1* ^−/−^ zebrafish, to establish whether NOD1 deficiency affects hatching process and larvae survival in the early ontogenesis, and to determine the possible molecular mechanisms. In addition, we conducted rescue experiments to investigate the correlation between NOD1 and CD44 receptors. The current study highlights NOD1 is essential for CD44a-mediated activation of the PI3K-Akt pathway and zebrafish larval survival.

## Results

### Generation of NOD1 knockout zebrafish using the Cas9/gRNA system

**Figure 1:**
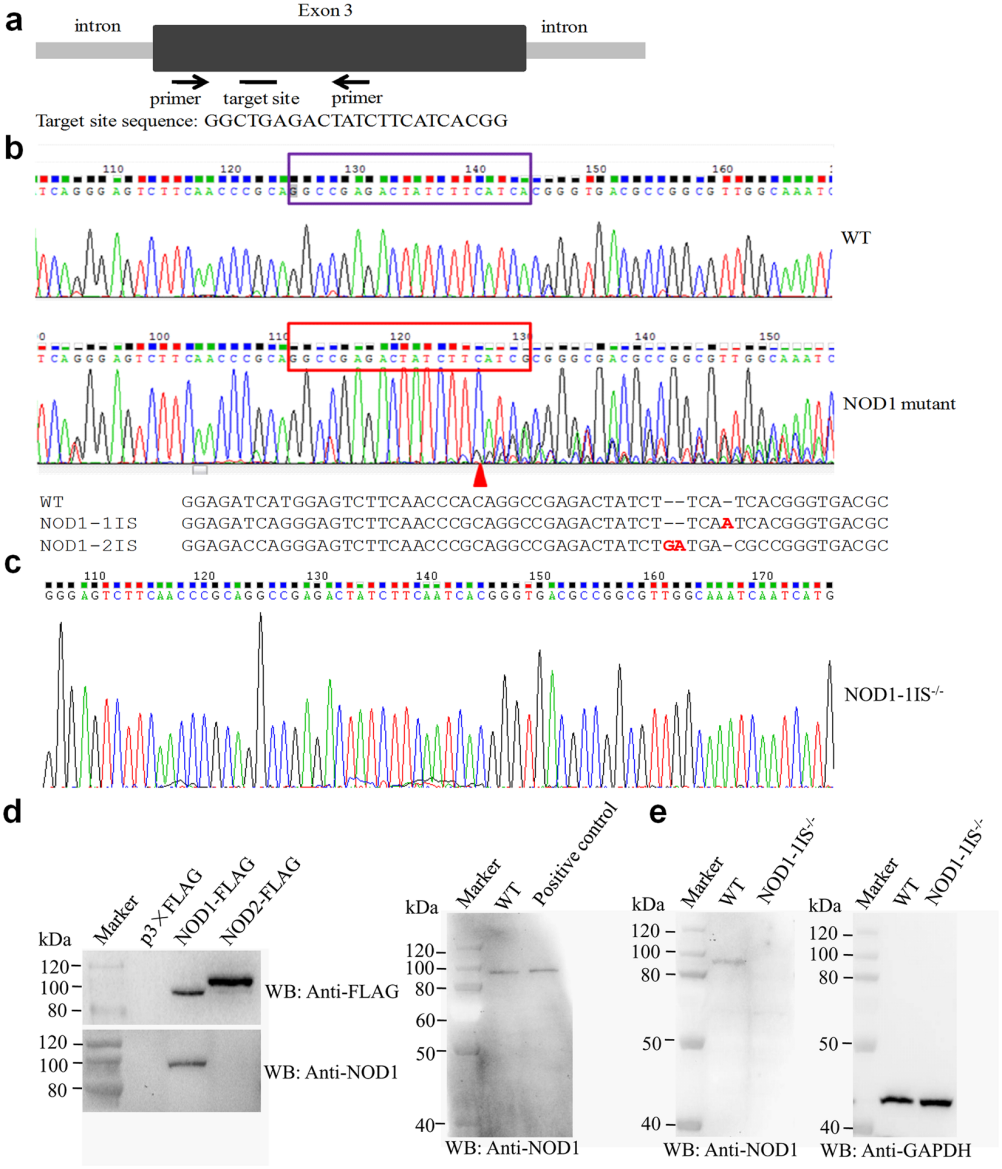
Cas9/gRNA induces indels in the NOD1 locus in zebrafish. (**a**) Cartoon showing the position of the target site and its sequence in the NOD1 locus in zebrafish. (**b**) Representative Sanger sequencing results of the PCR amplicons from the adult zebrafish induced by Cas9/ gRNA in the targeted NOD1 locus. PCR fragments were amplified and cloned into the pMD18-T vector from an individual zebrafish for Sanger sequencing. Insertion is highlighted in red. (**c**) Representative Sanger sequencing results of the PCR amplicons from homozygous mutations with 1 bp (*NOD1-1IS* ^−/−^). (**d**) NOD1 antibody specificity. Specificity of NOD1 monoclonal antibody was determined by immunoblot analysis of exogenous (left) and endogenous protein with the protein size of NOD1-FLAG as positive control (right). The lane labelled with p3 × FLAG is used as negative control, whose protein sample is from EPC cells transfected with p3 × FLAG empty plasmid. (**e**) NOD1 expression in wild-type (WT) zebrafish and homozygous mutations with 1 bp insertion (*NOD1-1IS* ^−/−^). Knockout of NOD1 was examined by analyzing NOD1 expression using immunoblotting with NOD1 monoclonal antibody in the WT and *NOD1-1IS* ^−/−^ larvae at 10 dpf.

Previous studies have shown that the Cas9/gRNA system efficiently executes site-specific cleavage, and is a highly effective and scalable gene knockout method in zebrafish *in vivo* ^38, 39^. To study the function of NOD1, we employed the Cas9/gRNA system to generate NOD1 knockout zebrafish. A gRNA with 23-bp “target sequence” (Fig. 1a) was designed, which starts with two GG residues at the 5′ end for efficient transcription from the T7 promoter and ends with the protospacer adjacent motif (PAM) NGG at the 3′ end, which is indispensable for Cas9 binding and cleavage^40^. Cas9 mRNA and NOD1 gRNA were microinjected into one-cell embryos of zebrafish. The results from sequencing of PCR fragments from a single zebrafish about 2 months old revealed two or more peaks at the same location. As expected, the Cas9/gRNA-mediated mutations occurred at or near the target site (Fig. 1b). A group of representative mutations was presented in Fig. 1b, including insertions of 1-2 base-pairs (*NOD1-1IS* ^−/−^ and *NOD1-2IS* ^−/−^). We further produced homozygotic *NOD1-1IS* ^−/−^ mutants through self-fertilization of heterozygotes that was confirmed by sequencing (Fig. 1c).

To confirm the deletion of NOD1 in zebrafish by western blotting, we generated an anti-NOD1 monoclonal antibody. Antibody specificity was verified by immunoblotting against transfected NOD1-FLAG or another NLR protein NOD2-FLAG. NOD1 antibody detected a strong band corresponding to the exogenous FLAG-tagged NOD1 (left in Fig. 1d). Using the NOD1-FLAG construct as a positive control, we detected a protein of similar size in wild-type (WT) zebrafish larvae. These results clearly demonstrate that NOD1 antibody specifically detect endogenous NOD1 protein in zebrafish (right in Fig. 1d). After confirming the specificity of zebrafish NOD1 antibody, we examined the effect of the *NOD1-1IS* ^−/−^ mutation on NOD1 protein expression by western blotting in NOD1 WT and knockout zebrafish larvae. The band corresponding to WT NOD1 protein (of ~100 kDa) was not detected in homozygous *NOD1-1IS* ^−/−^ mutants (left in Fig. 1e). This indicates that the Cas9/gRNA-mediated mutation ablates the expression of NOD1 in zebrafish.

### Loss of NOD1 in zebrafish impairs embryo hatching and larval survival

**Figure 2:**
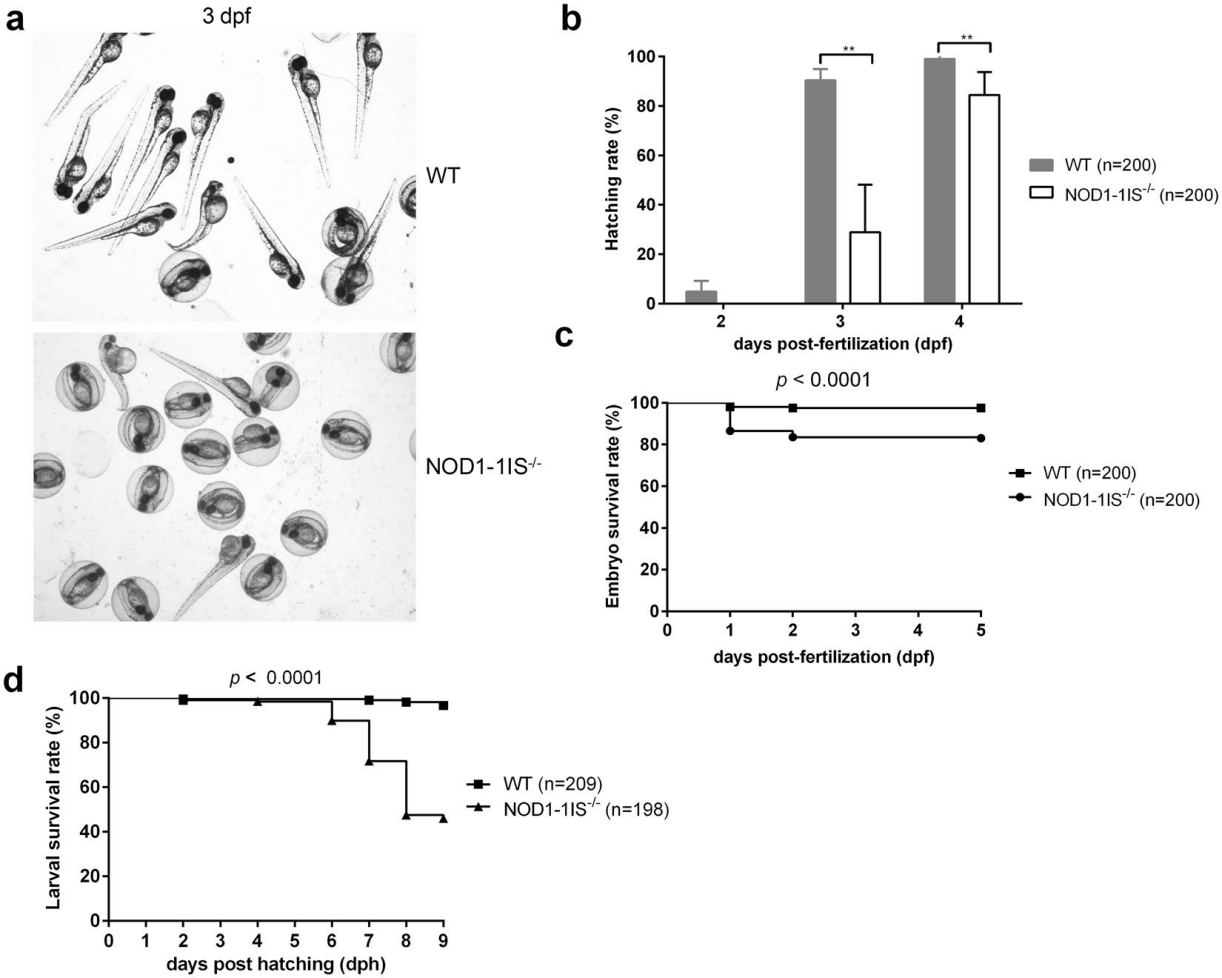
NOD1 is critical for embryo hatching and larvae survival. (**a**) The images of embryos and larvae from WT and *NOD1-1IS* ^−/−^ zebrafish at 3 days post-fertilisation (dpf). (**b**) The hatching rate of WT and *NOD1-1IS* ^−/−^ zebrafish at 2, 3 and 4 dpf (n = 4, each with 50 embryos). Data represent the mean ± the SEM, and were tested for statistical significance using two-tailed Student’s t-test. **p < 0.01. (**c**) The embryo survival curves of WT and *NOD1-1IS* ^−/−^ zebrafish (n = 4, each with 50 embryos). (**d**) The larval survival curves of WT and *NOD1-1IS* ^−/−^ zebrafish (n = 4, each with 49~52 larvae). The survival curves were compared statistically significant difference using the Log-Rank Test.

Our previous report concerning the constitutive expression of NOD throughout zebrafish development^37^ suggests that NOD genes have important functions in developmental processes. Hatching is a critical period for zebrafish embryogenesis. We thus evaluate the effect of NOD1 in zebrafish hatching process. At 2 days post-fertilization (dpf), only 4.9% of the WT zebrafish embryos hatched out of the chorion, and no statistical difference existed between the WT and *NOD1-1IS* ^−/−^ zebrafish. At 3 dpf, over 90% of the WT zebrafish embryos hatched out of the chorion, whereas *NOD1-1IS* ^−/−^ zebrafish embryos hatched at a significantly decreased rate of 28.9% (Fig. 2a and b). At 4 dpf, all the WT and *NOD1-1IS* ^−/−^ zebrafish embryos hatched out of the chorion, and the hatching rate increased to 84.4% in the *NOD1-1IS* ^−/−^ zebrafish embryos, indicating a delayed hatching process (Fig. 2b). Therefore, the pivotal period of NOD1 affecting embryo hatching happened at 3 and 4 dpf. Notably, NOD1 deficiency also impacts the embryo survival rate, and the deaths occurred mainly within the first day after fertilization (Fig. 2c).

The hatched larvae from WT and *NOD1-1IS* ^−/−^ zebrafish were used for survival analysis. We found that survival of the *NOD1-1IS* ^−/−^ zebrafish, compared to WT zebrafish, was similar during 1~5 days post-hatching (dph), slightly decreased at 6 dph, and greatly reduced at 7~9 dph (Fig. 2d). The death of *NOD1-1IS* ^−/−^ zebrafish peaked roughly at 5~8 dph. Overall, the survival rate of *NOD1-1IS* ^−/−^ zebrafish is significantly lower than that of WT zebrafish (Fig. 2d). Taken together, our results show that NOD1 deficiency in zebrafish impairs embryo hatching and larvae survival.

### Comparative transcriptome analysis reveals that NOD1 deficiency affects larval metabolism and immune system processes

**Figure 3:**
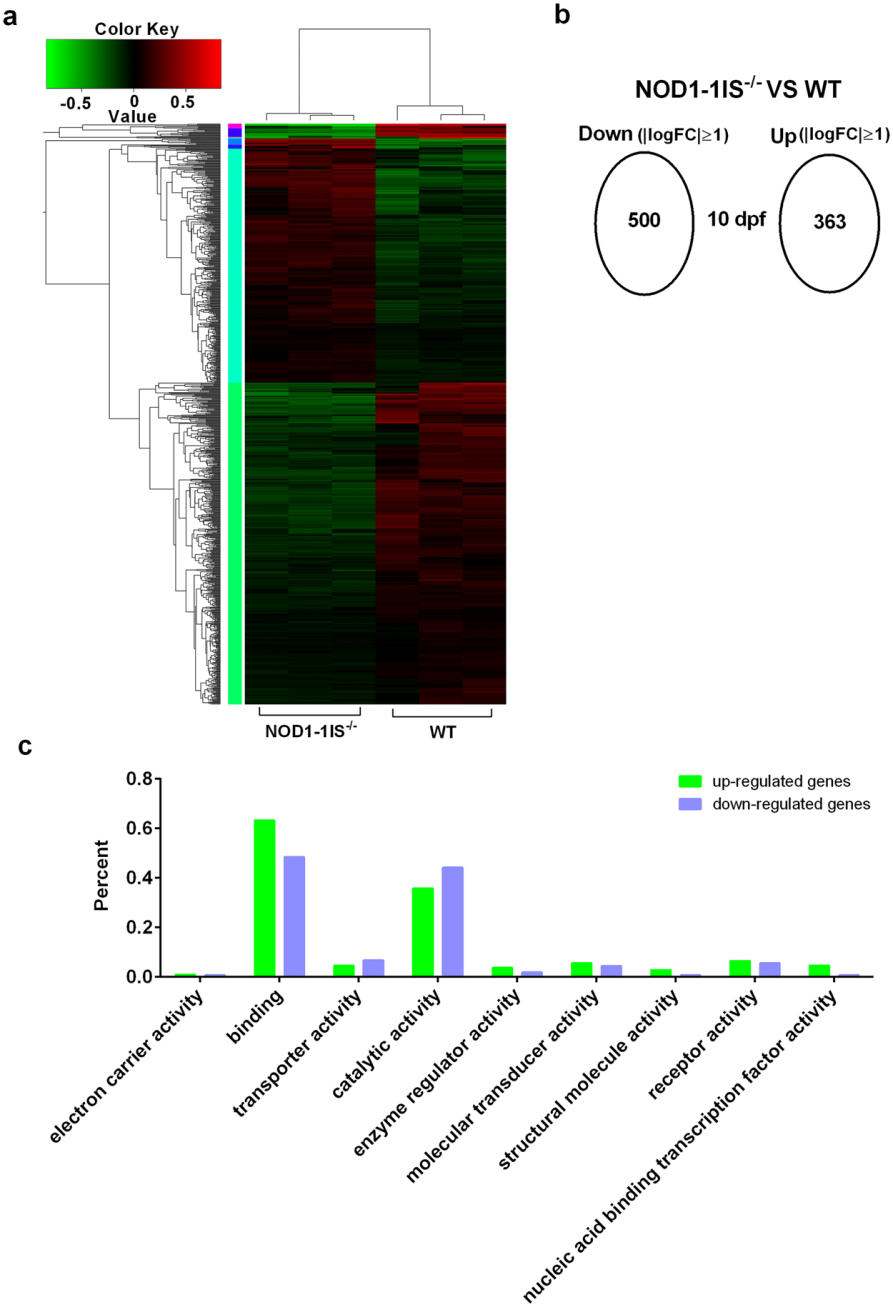
RNAseq profiling in WT vs *NOD1-1IS* ^−/−^ zebrafish larvae. (**a**) Heatmap analysis for transcriptomic data from the WT and *NOD1-1IS* ^−/−^ zebrafish larvae collected at 10 dpf. (**b**) Overview of transcriptomic data. (**c**) The molecular function category for differentially expressed genes.

To investigate the possible mechanism that NOD1 impacts larval survival in zebrafish, we performed transcriptome analysis to explore NOD1-related signaling pathways. Zebrafish larvae from WT and *NOD1-1IS* ^−/−^ were collected at 10 dpf (7 dph), when the survival rate of WT and *NOD1-1IS* ^−/−^ zebrafish larvae started to diverge significantly. The results showed that a total of 863 out of 40137 genes were considered to be differentially expressed (363 upregulated and 500 downregulated) when *p* value ≦ 0.05 and |logFC| ≧ 1 were set as the cut-off limits (Fig. 3a and b). Based on NR annotations, the Gene Ontology (GO) classification system was used to classify the possible functions of differentially expressed genes. A total of 272 genes (31.52%) were successfully assigned to at least one GO term annotation. For the molecular function category, the top two largest categories were “binding” and “catalytic activity” (Fig. 3c). We also performed an enrichment analysis of the KEGG pathways for all differentially expressed genes. The significantly enriched pathways were mainly involved in metabolism including “Metabolism of other amino acids”, “Xenobiotics biodegradation and metabolism”, “Lipid metabolism”, “Metabolism of cofactors and vitamins”, “Energy metabolism”, and involved in immune system including “Antigen processing and presentation” and “NOD-like receptor signaling pathway” (Supplementary Table S1).

**Figure 4:**
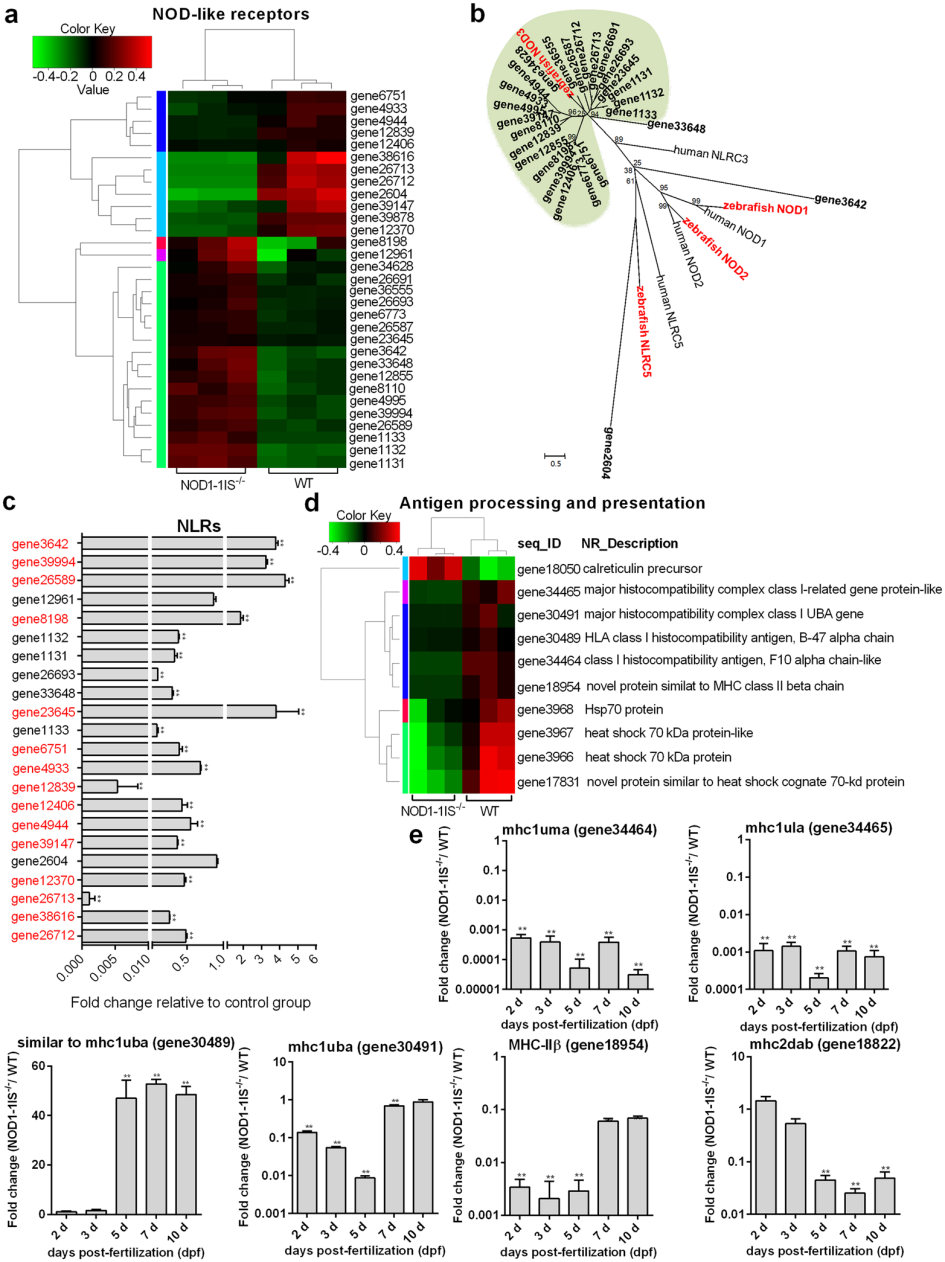
NOD1 is critical for innate and adaptive immunity during larval development. (**a**) The gene cluster for “NOD-like receptors”. At the top of this panel, a color key denotes the gradient scale of gene expression from low (green) to high (red) degrees. (**b**) Phylogenetic tree of NOD-like receptors. Phylogenetic relationships were based on amino acid alignments. Bootstrap values based on 10,000 replicates are indicated on each branch. The evolutionary history was inferred using the neighbor joining method. All positions containing gaps and missing data were eliminated from the dataset (pairwise deletion). Accession numbers of other NLR sequences from NCBI databases are as follows: zebrafish NOD2, NP_001314973; zebrafish NOD3, XP_003201049; zebrafish NLRC5, XP_003200494; human NLRC3, ACP40993; human NLRC5, NP_115582; human NOD1, AAD28350; human NOD2, AAG33677. (**c**) Validation of transcriptome sequencing results by quantitative RT-PCR. Those genes with the same expression tendency between RNA-seq and qRT-PCR are in red. Data represent the mean ± the SEM, and were tested for statistical significance using two-tailed Student’s t-test. **p < 0.01. (**d**) The gene cluster for “Antigen processing and presentation”. At the top of this panel, a color key denotes the gradient scale of gene expression from low (green) to high (red) degrees. (**e**) The expression changes of MHC class I and II genes in the *NOD1-1IS* ^−/−^ zebrafish compared with WT. 30~50 embryos or larvae from WT and *NOD1-1IS* ^−/−^ zebrafish were collected at 2, 3, 5, 7 and 10 dpf, and used for qRT-PCR. Data represent the mean ± the SEM, and were tested for statistical significance using two-tailed Student’s t-test. **p < 0.01.

Comparative transcriptome analysis showed that 31 NLRs containing NACHT and LRR domains were differentially regulated by NOD1 (Fig. 4a). Since the expansion of NLR-encoding genes has been described in the zebrafish^41, 42^, we further examined the phylogenetic relationship of these differentially expressed NLRs and NODs subfamily. The result of phylogenetic analysis showed these differentially expressed NLRs regulated by NOD1 were mainly the homologues of NOD3 (Fig. 4b). 22 NLR genes were also selected and used for qRT-PCR validation. PCR amplification showed that all qRT-PCR primers produced single fragments of the expected lengths (109~410 bp). Except for gene2604, gene1133, gene33648, gene26693, gene1131, gene1132 and gene12961, the expression of other 15 NLRs was in agreement with their transcript abundance changes determined by RNA-seq (Fig. 4c and a). Some data is not very consistent between RNA-seq and qRT-PCR, such as gene1133 and gene26693, which may be due to different batch samples or different calculation method for measuring gene expression. Besides NLRs, NOD1 also regulated the expression of tripartite motif proteins (15 DEGs) and immunity-associated proteins (17 DEGs) (Supplementary Fig. S1).

Comparative transcriptome analysis showed that a total of 10 genes involved in “Antigen processing and presentation” pathway, which include *calreticulin* (gene18050), *major histocompatibility complex class I-related gene protein-like* (gene34465), *major histocompatibility complex class I UBA gene* (gene30491), *HLA class I histocompatibility antigen, B-47 alpha chain* (gene30489), *class I histocompatibility antigen, F10 alpha chain-like isoform X1* (gene34464), *novel protein similat to MHC class II beta chain* (gene18954) and 4 *Hsp70* genes (gene3968, gene3967, gene3966 and gene17831), were differentially regulated by NOD1 (Fig. 4d). Except for *calreticulin*, the expression levels of other 9 DEGs were significantly weakened in *NOD1-1IS* ^−/−^ zebrafish larvae.

Although adaptive immunity is absent in the early zebrafish embryo and not functionally mature until at least 2 to 3 weeks postfertilization^43^, zebrafish larvae within 10 dpf have a constitutive expression for the above mentioned 5 MHC related genes and *mhc2dab* (gene18822)^44^. To further confirm the transcriptional regulation of NOD1 on the MHC class I and II genes, the expression patterns of 6 selected MHC related genes were furtherly studied in zebrafish embryo and larvae by qRT-PCR. Unexpectedly, the expression of *similar to mhc1uba* (gene30489) was up-regulated at *NOD1-1IS* ^−/−^ zebrafish larvae, whereas the expression of *mhc1uma* (gene34464), *mhc1ula* (gene34465), *mhc1uba* (gene30491), *MHC-IIβ* (gene18954) and *mhc2dab* (gene18822) were greatly attenuated at *NOD1-1IS* ^−/−^ zebrafish embryo and/ or larvae (Fig. 4e). These results show that NOD1 regulates the expression of MHC class I and II genes involved in “Antigen processing and presentation”.

Previous study showed that mammalian NOD1 was critically involved in the activation of NF-κB and MAPK^8^. However, comparative transcriptome analysis demonstrated that the conventional NF-κB and MAPK immune signaling pathways were normal in the *NOD1-1IS* ^−/−^ zebrafish larvae. To confirm the unchanged activities of NF-κB and MAPK, we performed qRT-PCR for NF-κB and MAPK target genes by using RNA from WT and *NOD1-1IS* ^−/−^ zebrafish larvae at 10 dpf. Most target genes including *nfkb1*, *nfkbiaa*, *nfkbiab*, *MAPK1*, *MAPK7*, *MAPK8*, *MAPK10* and *MAPK14* remained unchanged, except for the decreased expression of *nfkb2* and *MAPK3* (Supplementary Fig. S2). These results show that NOD1 deficiency did not significantly affect NF-κB and MAPK pathways at the transcriptional level.

### PCR arrays and western blotting reveal that NOD1 is critical for CD44a/Lck as well as PI3K/Akt pathway

**Figure 5:**
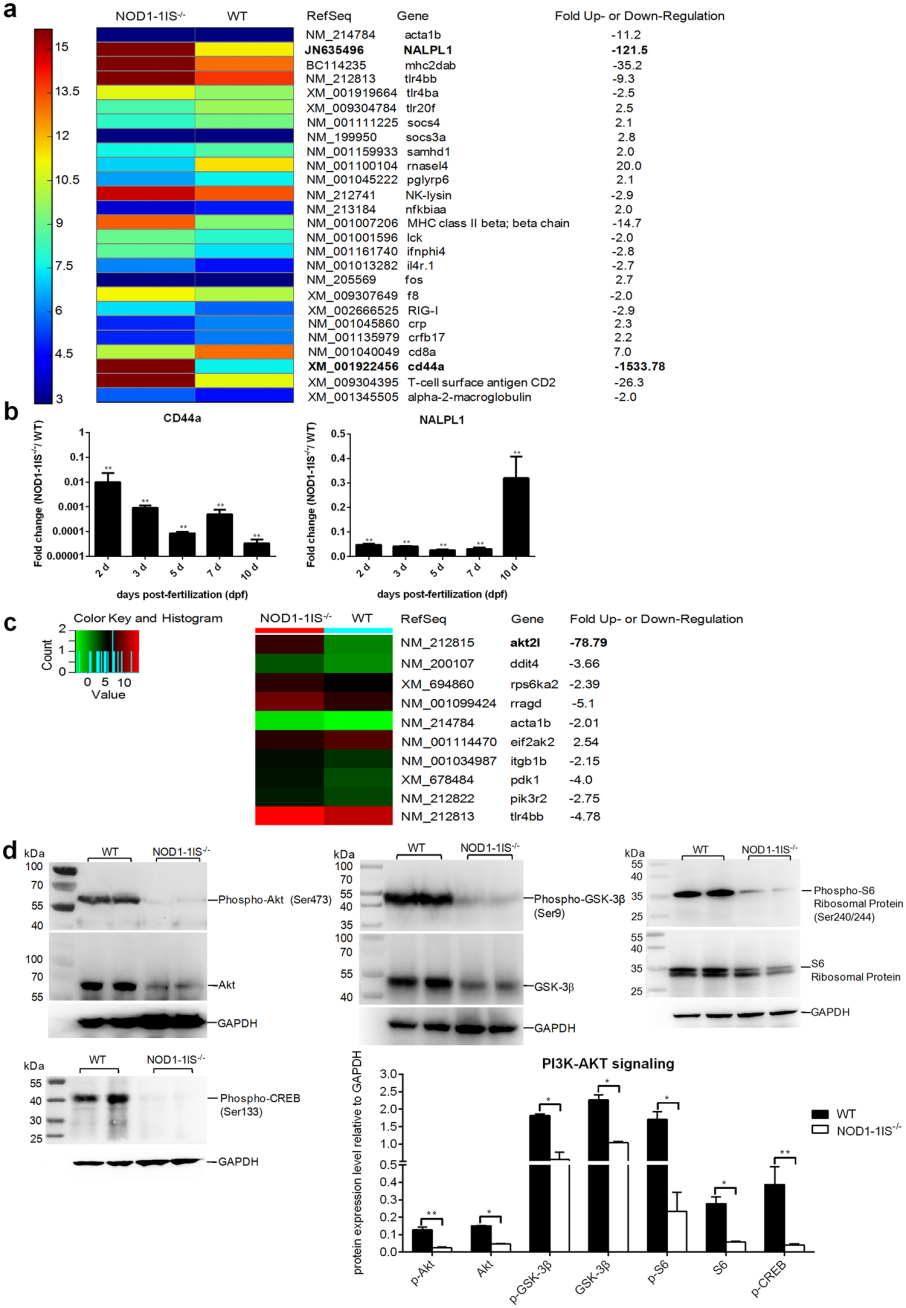
NOD1 deficiency impairs CD44a-PI3K-Akt pathway. (**a**) Heatmap analysis for PCR-arrays data from WT and *NOD1-1IS* ^−/−^ zebrafish at 10 dpf. The custom RT^﻿2﻿^ Profiler PCR arrays containing 372 genes involved in Type I interferon response, Toll-like receptor signaling, JAK/ STAT signaling, T-Cell & B-cell activation, antibacterial and antiviral responses were applied. Three biological replicates were performed for the WT and *NOD1-1IS* ^−/−^ zebrafish. (**b**) The expression changes of CD44a and NALPL1 in the *NOD1-1IS* ^−/−^ zebrafish compared with WT. 30~50 embryos or larvae from WT and *NOD1-1IS* ^−/−^ zebrafish were collected at 2, 3, 5, 7 and 10 dpf, and used for qRT-PCR. Data represent the mean ± the SEM, and were tested for statistical significance using two-tailed Student’s t-test. **p < 0.01. (**c**) Heatmap analysis for PCR-arrays data from WT and *NOD1-1IS* ^−/−^ zebrafish at 10 dpf. *Zebrafish PI3K-Akt Signaling Pathway RT* ^2^ *Profiler PCR Array* and *mTOR Signaling RT² Profiler™ PCR Array* were applied. Three biological replicates were performed for the WT and *NOD1-1IS* ^−/−^ zebrafish. (**d**) Immunoblot analysis of phospho-Akt, Akt, phospho-S6, S6, phospho-GSK3β, GSK3β and phospho-CREB in larvae homogenate from WT and *NOD1-1IS* ^−/−^ zebrafish at 10 dpf. Western blotting results were quantified using Quantity One software. Data represent the average of two independent experiments. *p < 0.05, **p < 0.01.

The above results indicate that NOD1 deficiency affects larval immune system. Since 32% inconsistent expression exists between RNA-seq and qRT-PCR, we further validate the expression difference of innate and adaptive immune genes between WT and *NOD1-1IS* ^−/−^ zebrafish collected at 10 dpf by our previous custom RT^2^ Profiler PCR arrays^44^. This array contains a total of 372 genes involved in Type I interferon response, Toll-like receptor signaling, JAK/ STAT signaling, T-Cell & B-cell activation, antibacterial and antiviral responses. The array analysis revealed that 11 genes were up-regulated and 15 genes were down-regulated in *NOD1-1IS* ^−/−^ zebrafish. Among them, *NALPL1* and *CD44a* were the top two down-regulated in *NOD1-1IS* ^−/−^ zebrafish, with a decrease in expression of 122- and 1534-fold, respectively (Fig. 5a). The tyrosine kinase *Lck*, which associated with *CD44*, was also down-regulated in *NOD1-1IS* ^−/−^ zebrafish (Fig. 5a). Since the expression tendency of *NALPL1* and *CD44a* from PCR arrays was inconsistent with transcriptome data, we further examined the expression of *NALPL1* and *CD44a* at more time points with more biological replicates. The results of qRT-PCR showed that NOD1 deficiency did impair the expression of *NALPL1* and *CD44a* both at zebrafish embryo and larvae (Fig. 5b).

Mammalian CD44 is a widely-expressed adhesion receptor, and involved in activating the PI3K/Akt signaling pathway, which is associated with cell survival in various cell types^33^. Comparative transcriptome analysis showed that 11 genes involved in PI3K/Akt signaling pathway were differentially regulated by NOD1 (Supplementary Fig. S3a). To determine whether NOD1 impacts larval survival through CD44a-mediated modulation of the PI3K/Akt signaling, we evaluated the effect of NOD1 on the activation of PI3K and Akt downstream of mTOR. We employed two PCR arrays to profile the PI3K-Akt and mTOR signaling cascades. Zebrafish larvae from WT and *NOD1-1IS* ^−/−^ collected at 10 dpf were used for *Zebrafish PI3K-Akt Signaling Pathway RT* ^2^ *Profiler PCR Array* and *mTOR Signaling RT² Profiler™ PCR Array*. Among 84 genes involved in the PI3K-Akt pathway, the expressions of *pdk1*, *tlr4bb*, *itgb1b* and *PIK3R2* were significantly decreased, whereas that of *eif2ak2* was significantly increased in *NOD1-1IS* ^−/−^ zebrafish compared to WT zebrafish. Within the 84 genes involved in the mTOR pathway, transcripts of *akt2l*, *ddit4*, *rps6ka2* and *rragd* were reduced in *NOD1-1IS* ^−/−^ zebrafish, with that of *akt2l* was the most pronounced (79-fold) (Fig. 5c). To further corroborate with the gene induction of these two pathways, we probed the protein expressions of Akt and several key substrates thereof, including GSK3β, S6 and CREB, by western blotting. The result demonstrated that loss of NOD1 inhibited the expression of Akt, GSK3β and S6. The phosphorylation levels of Akt, GSK3β, S6 and CREB were also decreased in *NOD1-1IS* ^−/−^ zebrafish collected at 10 dpf (Fig. 5d). The decreased levels of Akt and phosphorylated GSK-3β were observed at earlier time points such as 5 and 7 dpf. The expression of p-Akt, GSK3β and S6 in *NOD1-1IS* ^−/−^ zebrafish was also decreased at 7 dpf compared with WT zebrafish (Supplementary Fig. S3b). These results indicate that loss of NOD1 results in the inhibition of PI3K-Akt signaling.

### CD44a is critical for NOD1-mediated regulation of PI3K-Akt, but not for NOD1-mediated regulation of MHC class I and II genes

**Figure 6:**
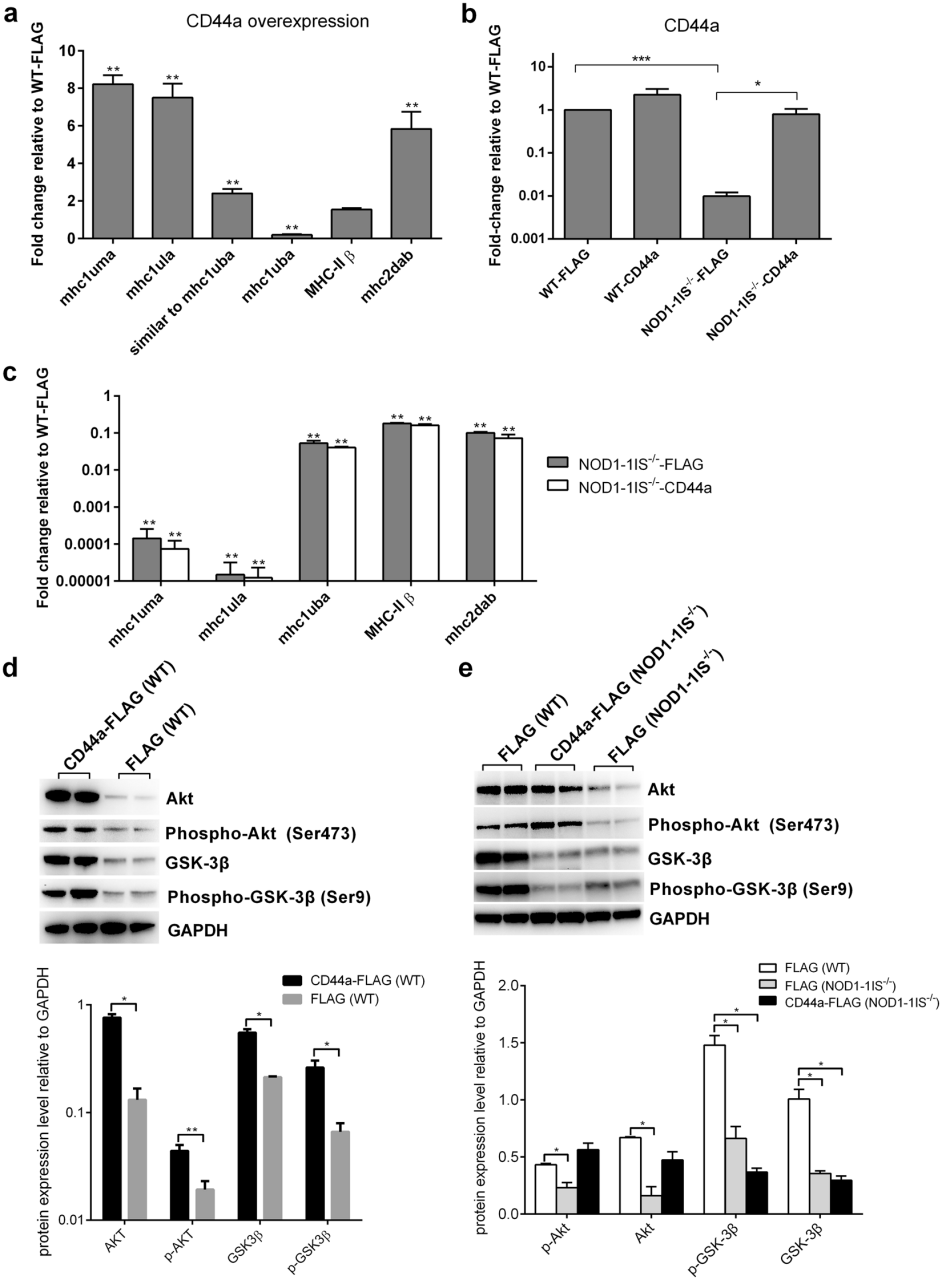
NOD1 regulates Akt expression through CD44a. (**a**) The effect of CD44a overexpression in WT zebrafish on the expression of MHC class I and II genes. (**b**) The expression of CD44a in WT and *NOD1-1IS* ^−/−^ zebrafish microinjected with p3 × FLAG or CD44a-FLAG. (**c**) The expression of MHC class I and II genes in *NOD1-1IS* ^−/−^ zebrafish. For (a-c), all samples were collected at 7 dpf and used for RNA extraction and qRT-PCR (mean ± SEM, n = 3). The WT zebrafish microinjected with p3 × FLAG were used as control. Data were tested for statistical significance using two-tailed Student’s t-test. ***p < 0.001, **p < 0.01,*p < 0.05. (**d**) Immunoblot analysis of Akt, GSK-3β, phospho-Akt and phospho-GSK-3β in larvae homogenate from WT zebrafish microinjected with p3 × FLAG or CD44a-FLAG (n = 50~60 larvae each group) at 7 dpf. (**e**) Immunoblot analysis of Akt, GSK-3β, phospho-Akt and phospho-GSK-3β in larvae homogenate from WT and *NOD1-1IS* ^−/−^ zebrafish at 7 dpf microinjected with p3 × FLAG or CD44a-FLAG (n = 50~60 larvae each group). For (d-e), western blotting results were quantified using Quantity One software. Data represent the average of two independent experiments. *p < 0.05, **p < 0.01.

During embryonic and larval development, many MHC class I and class II molecules, such as *mhc1uma*, *mhc1ula* and *MHC-IIβ*, have similar impaired expression pattern with *CD44a* in *NOD1-1IS* ^−/−^ zebrafish (Fig. 4e and Fig. 5b). Further, the overexpression of *CD44a* in WT zebrafish could induce the expression of these MHC class I and class II molecules, such as *mhc1uma*, *mhc1ula*, *similar to mhc1uba* and *mhc2dab* (Fig. 6a). To determine whether *CD44a* is required for the defective expression of MHC related genes in *NOD1-1IS* ^−/−^ zebrafish, rescue experiments were performed by overexpression of *CD44a* in NOD1 knockout zebrafish. The impaired expression of endogenous *CD44a* in *NOD1-1IS* ^−/−^ zebrafish could be rescued by exogenous *CD44a* (Fig. 6b). However, the expression of exogenous *CD44a* failed to restore the expression of MHC related genes in *NOD1-1IS* ^−/−^ zebrafish (Fig. 6c). These results show that *CD44a* expression is not sufficient for NOD1-mediated MHC genes expression, suggesting that other unknown signaling pathways are required for MHC gene expression downstream of NOD1.

We next tested whether the decreased PI3K-Akt signaling in *NOD1-1IS* ^−/−^ zebrafish was due to the impaired expression of CD44a. Overexpression of *CD44a* was found to be effective until 7 dpf (13-fold, Supplementary Fig. S4a) and could restore *CD44a* expression in *NOD1-1IS* ^−/−^ zebrafish at 7 dpf (Fig. 6b), we thus examined the PI3K-Akt signaling cascade from WT and NOD1 knockout zebrafish with or without CD44a overexpression at 7 dpf by western blotting. In WT zebrafish, CD44a overexpression increased the levels of phosphorylated and total proteins of Akt and GSK3β (Fig. 6d). Furthermore, overexpression of CD44a in *NOD1-1IS* ^−/−^ zebrafish rescued the expression of phosphorylated and unphosphorylated Akt, but not for GSK3β, an enzyme that regulated glycogen synthesis (Fig. 6e). Taken together, these results suggest that NOD1 regulates Akt expression through CD44a.

### CD44a overexpression in *NOD1-1IS*^−/−^ zebrafish rescues larval survival

**Figure 7:**
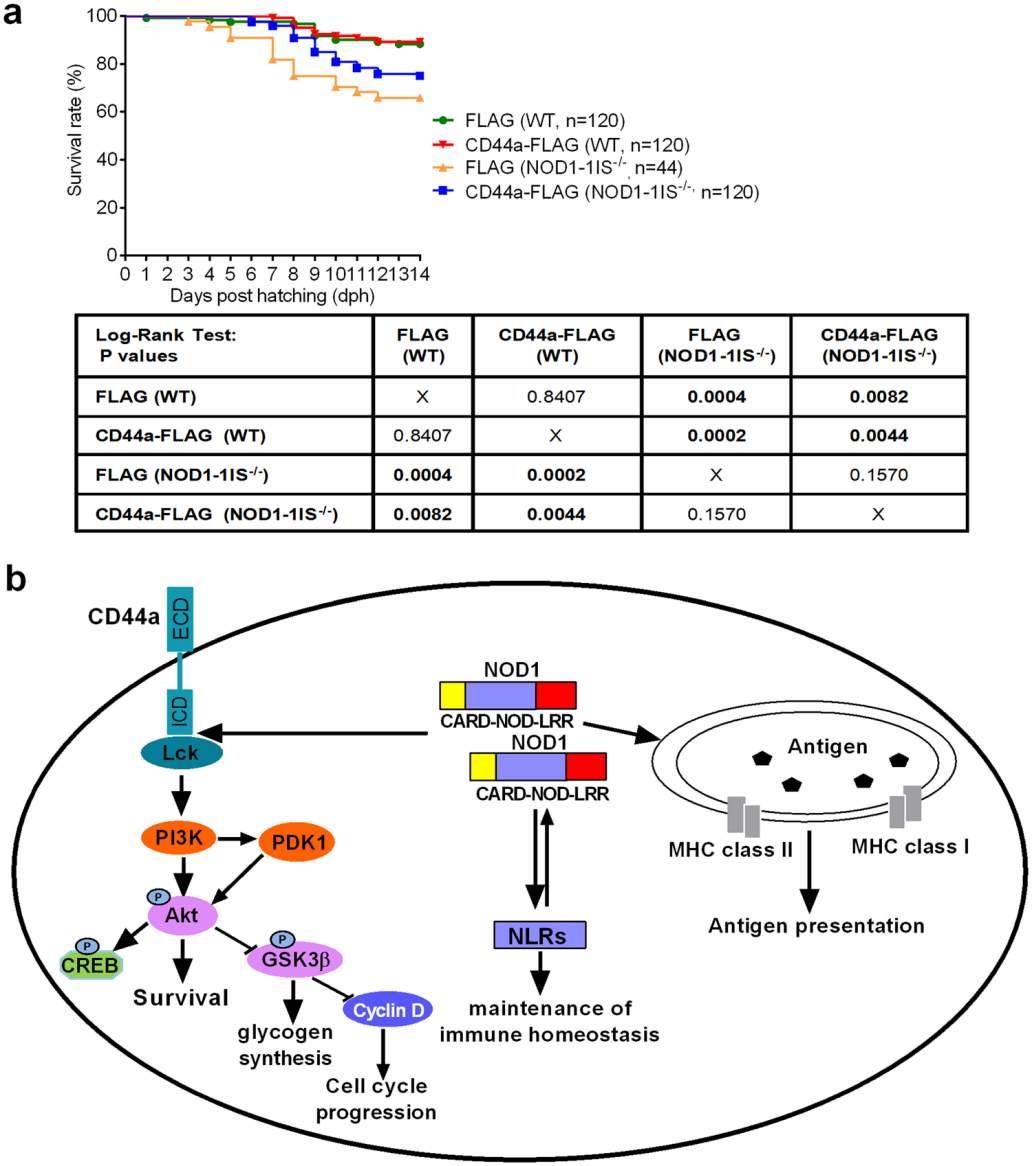
CD44a overexpression in *NOD1-1IS* ^−/−^ zebrafish rescues larval survival. (**a**) Survival of WT and *NOD1-1IS* ^−/−^ zebrafish microinjected with p3 × FLAG or CD44a- FLAG (n = 44~120 larvae each group). The survival curves lasting for 14 dph were compared statistically significant difference using the Log-Rank Test. (**b**) Proposed model illustrating the intracellular signaling pathways modulated by NOD1 during zebrafish larval development at homeostasis. p, phosphorylation.

Having shown that CD44a overexpression rescued Akt expression in *NOD1-1IS* ^−/−^ zebrafish, we wanted to know whether CD44a overexpression could rescue larval survival. The hatched larvae from WT and *NOD1-1IS* ^−/−^ zebrafish microinjected with control or CD44a-FLAG construct were used for survival analysis. Compared with WT zebrafish microinjected with the control plasmid, no obvious difference was observed for WT zebrafish microinjected with the CD44a-FLAG plasmid up to 14 dph (*p* = 0.8407), and significant divergence of survival curves observed for *NOD1-1IS* ^−/−^ zebrafish microinjected with the control (*p* = 0.0004) or CD44a-FLAG construct (*p* = 0.0082) (Fig. 7a). Since we noted that CD44a was not sufficiently overexpressed in zebrafish larvae at 12 dpf (corresponding to 8 dph), a statistically significant difference of the survival curves lasting for 8 dph were again observed using the Log-Rank Test. As shown in Supplementary Fig. S4b, no significant divergence of survival curve was observed between WT zebrafish microinjected with the control plasmid and *NOD1-1IS* ^−/−^ zebrafish microinjected with the CD44a-FLAG construct (*p* = 0.0683). However, *NOD1-1IS* ^−/−^ zebrafish microinjected with CD44a-FLAG had a higher survival rate than *NOD1-1IS* ^−/−^ zebrafish microinjected with the control construct, with the observed significance level (*p* = 0.0056) (Supplementary Fig. S4b). This result shows that NOD1 impacts larvae survival via CD44a.

## Discussion

Although the *in vivo* relevance of NOD1-mediated signaling for immunity against various pathogens including bacteria, virus and parasites has been clearly demonstrated^9, 45, 46^, the role of NOD1 during developmental processes has not been explored in detail. In the present study, we demonstrate that zebrafish NOD1 is required for hatching process and larval survival. The present study demonstrates that NOD1 is a multifunctional regulator that drives the expression of multiple receptors and immune signaling pathways. The present study also confirms the crucial role of NOD1 in larval survival through a CD44a-mediated PI3K-Akt signaling cascade.

Multiple studies have identified positive or negative regulatory functions of NLRs in innate immune responses. Studies of gene deletion or knockdown show that NLRP6 impedes the clearance of both Gram-positive and -negative bacterial pathogens through negatively regulating MAPK and canonical NF-κB pathways^47^, while NLRP12 is a negative regulator of inflammatory T cell responses and T cell-mediated disease^48^. NLRC3 negatively regulates innate immune signaling induced by the DNA sensor STING^49^. In line with afore mentioned studies, NLRC5 and NLRX1 attenuate innate immune responses by inhibiting the NF-κB and type I interferon pathways^50, 51^. NOD2 is necessary for the NF-kappaB/IL-1beta-mediated innate responses against bacteria challenge in Crohn’s disease^52^. These findings highlight a broad spectrum of functions of NLRs in regulating innate immune responses. MHC class I and II pathways play essential roles in the activation of adaptive immune responses. Among NLR members, NLRC5 and CIITA are key transcriptional regulators of MHC class I and II genes, respectively^53–56^. Interestingly, we showed that NOD1 is required for the expression of both MHC class I and II genes *in vivo*. The present study identified NOD1 as a new regulator to drive the expression of MHC class I and II genes. Further studies are needed to clarify the mechanism by which NOD1 transcriptionally regulates the expression of MHC class I and II genes, and if NOD1 plays redundant or exclusive roles with NLRC5 and CIITA in MHC class I- and class II-dependent immune responses.

NLRs, Toll-like receptors (TLRs) and RIG-I-like receptors (RLRs) are three families of pathogen sensors. Much progress has been made on how these innate sensors are activated to initiate host innate immune signaling. It has become increasingly apparent that there are extensive interplays among these families of pattern recognition receptors^57^. Various levels of crosstalk between NLRs and TLRs, and between NLRs and RLRs have been described^58, 59^. NOD1 and NALPL1 belong to the members of NLR family. Our previous work showed that overexpression of NALPL1 contributed to protection against bacterial and viral infection. Moreover, NALPL1 overexpression induced the expression of NOD1^60^. The impaired expression of NALPL1 in the *NOD1-1IS* ^−/−^ larvae reported here suggests that NALPL1 and NOD1 constitute a feed-forward circuit in host immune defense. Comparative transcriptome analysis also screened multiple differentially expressed NLRs regulated by NOD1. Our unpublished data showed that these NLRs regulated by NOD1 positively or negatively regulated innate immune signaling, which suggested that the interplays between NOD1 and other NLRs may contribute to the maintenance of immune homeostasis.

It is well accepted that CD44 is a major cell surface receptor for the extracellular matrix glycosaminoglycan hyaluronan, and serves as a coreceptor for other receptors such as epidermal growth factor receptor (EGFR)/ErbB1 and ErbB2/Her2^61^, c-Met^62^, TGFβ receptors 1 and 2^63^. CD44 prevents exaggerated inflammatory responses to LPS by promoting the expression of negative regulators of TLR4 signaling^64^. It also directly associates with TLR2 which negatively regulates TLR-mediated inflammatory responses^65^ and participates in IP-10 induction^35^. In addition, ligation of CD44 can activate the PI3K-Akt signaling pathway that is critical for cell survival of various cell types^33, 66, 67^. Therefore, it is significant to find the modulation of zebrafish NOD1 on the CD44a and PI3K-Akt signaling. Furthermore, the expression of exogenous CD44a in zebrafish embryos had no effect on NOD1 expression (Supplementary Fig. S4c). However, we found that the impaired expression of phosphorylated and unphosphorylated Akt could be rescued by CD44a overexpression in *NOD1-1IS* ^−/−^ larvae. Collectively, these findings suggest that NOD1 acts as an activator upstream of the CD44a-mediated PI3K-Akt signaling pathway. Since we can’t find a direct interaction between zebrafish NOD1 and CD44a, the intermediate signaling molecule linking NOD1 and CD44a-mediated PI3K-Akt signaling pathways will be the interest of future study.

In mammals, the phosphoinositide-3-kinase/protein kinase B/mammalian target of rapamycin (PI3K/Akt/mTOR) pathway is emerging as a critical integrator of intracellular signals, and important for apoptosis, malignant transformation, tumor progression, metastasis and radioresistance^68–71^. Due to the important role of the PI3K/Akt/mTOR pathway in growth control and cancer research^72–74^, many valuable inhibitors targeting in this pathway have been developed in recent years^75, 76^. NLRC3 (also known as CLR16.2 or NOD3), another member of the NLR Family, is recently identified as an inhibitory sensor of the PI3K/Akt/mTOR pathway, mediating protection against tumorigenesis in colorectal cancer^77^. In the present study, the finding that NOD1 is involved in the regulation of NLRC3 homologues, nutrient metabolism as well as the CD44a-mediated PI3K/Akt/mTOR pathway is very intriguing, which suggest the multiple functions of NOD1 in different field of biology including infectious disease, metabology, oncology, developmental biology, and not confined to the immunology.

In conclusion, the current study revealed the regulation of innate and adaptive immunity by NOD1 during zebrafish larval development. Based on the experimental data mentioned or discussed above and previous reports, a working model of how NOD1 affects intracellular signaling is shown in Fig. 7b. Most notably, the current study demonstrates that NOD1 acts as an activator upstream of the CD44a-mediated PI3K-Akt signaling pathway in larval survival. Future studies will focus on the mechanism by which NOD1 transactivates MHC class I and II related genes, and the effect of the correlation between NOD1 and CD44 signaling in pathogen infection.

## Methods

### Zebrafish care and maintenance

*NOD1-1IS* ^−/−^ zebrafish were obtained from the China Zebrafish Resource Center (CZRC). Wild-type AB/TU and mutant zebrafish were raised and maintained under standard conditions^78^. Zebrafish embryos were obtained by artificial insemination and reared at 28 °C. All animal experiments were conducted in accordance with the Guiding Principles for the Care and Use of Laboratory Animals and were approved by the Institute of Hydrobiology, Chinese Academy of Sciences (Approval ID: IHB 2013724).

### Cell culture and transfection

Epithelioma papulosum cyprini (EPC) and zebrafish ZF4 (embryonic fibroblast cell line) cells were maintained in M199 and DMEM/F12 (1:1) medium supplemented with 10% FBS at 28 °C, respectively. Transfection of plasmids was performed using Lipofectamine 2000 (Invitrogen).

### Zebrafish embryo-larval assay

For the analysis of zebrafish hatching process, the fertilized eggs from the WT and *NOD1-1IS* ^−/−^ parent were randomly divided into four experimental groups, each with 50 embryos. Water was renewed every day in dishes. The hatched larvae were recorded at 2, 3 and 4 dpf to evaluate the hatching rate. For embryo survival analysis, the fertilized eggs from the WT and *NOD1-1IS* ^−/−^ parent were randomly divided into four experimental groups, each with 50 embryos. The dead embryos or larvae were recorded daily until 5 dpf.

For larval survival analysis, the larvae from the WT and *NOD1-1IS* ^−/−^ parent at 4 dpf were randomly divided into four groups, each with 49~52 larvae. Larvae survivals were recorded daily until 9 dph (12 dpf). Dead individuals were removed each day.

### cDNA library construction and Illumina deep sequencing

Total RNA was isolated from the WT and *NOD1-1IS* ^−/−^ zebrafish at 10 dpf using the TRIzol® Reagent (Invitrogen). The quantity and quality of total RNA were checked by Nanodrop2000 (Thermo Scientific) and Bioanalyzer 2100 (Agilent). Sequencing libraries for whole transcriptome analysis were generated using TruseqTM RNA sample prep Kit (Illumina). After quantitation using TBS380 Picogreen (Invitrogen), the libraries were sequenced using Hiseq4000 Truseq SBS Kit v3-HS(200cycles)(Illumina). To identify differentially expressed genes between the WT and *NOD1-1IS* ^−/−^ zebrafish, the expression levels were measured by using numbers of fragments per kilobase of transcript per million fragments sequenced (FPKM). The raw sequences were deposited at NCBI Sequence Read Archive (Accession No. SRP090717).

### PCR arrays and qRT-PCR

RNA used for PCR arrays was extracted from the WT and *NOD1-1IS* ^−/−^ zebrafish at 10 dpf using TRIzol reagent, and reverse transcribed to cDNA using SuperScript III Reverse Transcriptase (invitrogen). To study the expression profile of genes involved in various cellular pathways, pathway-specific PCR arrays including *zebrafish PI3K-Akt Signaling Pathway RT* ^2^ *Profiler PCR Array* (Qiagen), *zebrafish mTOR Signaling RT² Profiler™ PCR Array* (Qiagen) and custom RT^2^ Profiler PCR arrays (SABioscience) containing 372 innate and adaptive immune genes were used. Data was analyzed using the manufacturer’s software. Three biological replicates were performed for the WT and *NOD1-1IS* ^−/−^ zebrafish. PCR arrays data were deposited at www.ncbi.nlm.nih.gov/geo with the accession numbers GSE80621 and GSE80622.

For understanding the expression changes of CD44a, MHC class I and II genes during embryonic and larval development from *NOD1-1IS* ^−/−^ zebrafish, the effect of CD44a overexpression on the expression of NOD1, CD44a, MHC class I and II genes from WT zebrafish, and whether CD44a rescued the expression of MHC class I and II genes from *NOD1-1IS* ^−/−^ zebrafish, RNA was extracted using Trizol (Invitrogen) from snap-frozen embryos or larvae collected at different time points. CDNA was obtained using RevertAidTM First Strand cDNA Synthesis Kit (Fermentas) with a oligo(dT)_18_ primer. QRT-PCR was then performed on a BIO-RAD CFX96 Real-Time System using iQ™ SYBR^®^ Green Mix (BIO-RAD) according to manufacturer’s instructions. Quantifications were performed on triplicate wells. To normalize cDNA amounts, the housekeeping gene GAPDH was used. The primers specific for the gene of interest were listed in Supplementary Table S2.

### Western Blot analysis

To detect NOD1 antibody specificity, 1 × 10^6^ EPC cells overexpressing NOD1 and NOD2 were lysed in 200 μl cold Pierce^®^ IP lysis buffer (Prod# 87788) containing protease inhibitor (Prod# 1860932), then separated by SDS–PAGE, transferred to PVDF membranes and immunoblotted with the primary antibodies of monoclonal mouse anti-FLAG tag antibody (Sigma, F3165) and monoclonal mouse anti-NOD1 antibody at a dilution of 1:5000 and 1:1000, respectively. To detect endogenous NOD1 protein, 5 × 10^6^ ZF4 cells were lysed in 300 μl cold Pierce^®^ IP lysis buffer containing protease inhibitor. For NOD1 expression in WT and *NOD1-1IS* ^−/−^ zebrafish, 50 larvae at 10 dpf were lysed in 200 μl cold Pierce^®^ RIPA buffer (Prod# 89900) containing protease inhibitor using ultrasonication.

For detecting the protein expression involved in PI3K-Akt pathway, 60~250 larvae at 5, 7 and/or 10 dpf from WT and *NOD1-1IS* ^−/−^ zebrafish with or without the transfection of FLAG or CD44-FLAG were lysed in 200~800 μl cold RIPA buffer containing phosphatase inhibitor (Prod #78420) and protease inhibitor using ultrasonication. Primary antibodies used were phospho-Akt (Cell Signaling #4060 S), Akt (Cell Signaling #9272), phospho-GSK-3β (Cell Signaling #9323), GSK-3β (Cell Signaling #9315), phospho-S6 ribosomal protein (Cell Signaling #2215), S6 ribosomal protein (Cell Signaling #2217) and phospho-CREB (Cell Signaling #9198) at a dilution of 1:1000. Mouse monoclonal anti-GAPDH (proteitech, 60004-1-Ig) was used throughout as a loading control. Secondary antibodies were diluted 1:5000 including Pierce goat anti-rabbit IgG and goat anti-mouse IgG (Prod# 31460 and #31430). The bands were detected using Pierce ECL Western Blotting Substrate (Prod# 32106) and ECLWestern blot system (LAS-4000mini, Fuji, Japan) according to the manufacturer’s instructions. Densitometer analysis was performed using Quantity One software (BioRad).

### Rescue experiments

In the rescue experiments, 2-cell embryos from WT and *NOD1-1IS* ^−/−^ zebrafish were microinjected with FLAG or CD44-FLAG plasmid, which were diluted to the desired concentration of 100 ng/μl. The typical injected volume was 2 nl. The injected embryos were raised at 28 °C in fish water.

### Statistical analysis

Expression data by qRT-PCR and western blotting are presented as means and standard error of mean (SEM). Two-tailed Student’s t-test was used to compare statistical significance. All data are representative of two to four independent experiments. The level of significance is shown as follows: *, *p* < 0.05; **, *p* < 0.01, ***, *p* < 0.001. Significance testing in the cumulative survival analysis used Log Rank test using GraphPad Prism software.

### Data Availability

All data generated or analysed during this study are included in this published article and its Supplementary Information files.

## Electronic supplementary material

Supplementary Information.
