# Supplementary material for: NOD1 deficiency impairs CD44a/Lck as well as PI3K/Akt pathway
Source: Sci Rep. 2017 Jun 7;7:2979. doi: 10.1038/s41598-017-03258-y (PMC5462776; doi:10.1038/s41598-017-03258-y)
Supplement: Supplementary file 1 — Supplementary Information. [file 41598_2017_3258_MOESM1_ESM.pdf]

# NOD1 deficiency impairs CD44a/Lck as well as PI3K/Akt pathway

Yi Wei Hu, Xiao Man Wu, Shi Si Ren, Lu Cao, Pin Nie, Ming Xian Chang*

**Figure S1**

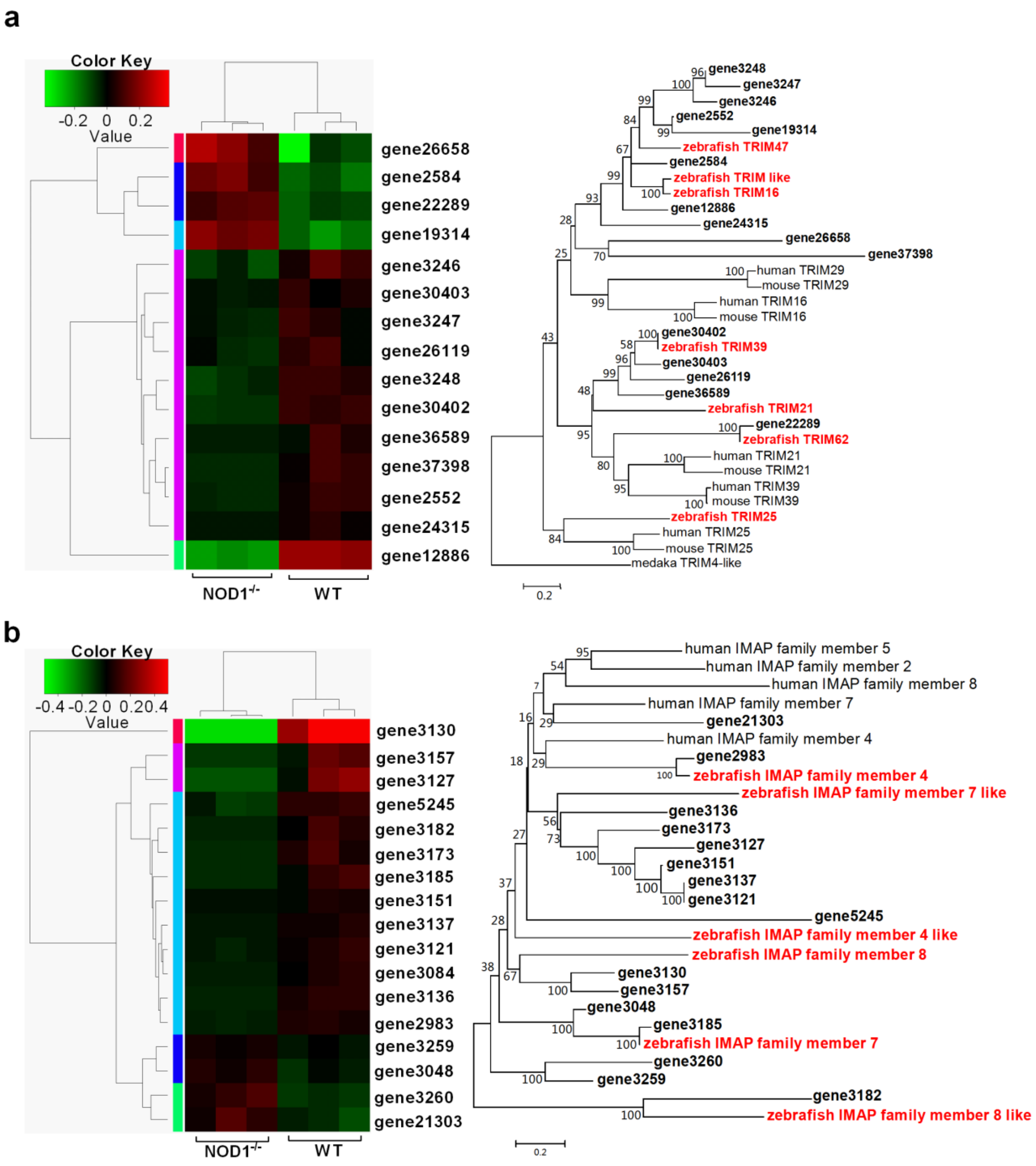


**Fig. S1. Heatmap and phylogenetic analysis for differentially expressed genes involved in immune system. (a)** Heatmap and phylogenetic analysis for tripartite motif proteins (TRIMs). Accession numbers of other TRIMs from NCBI database are as follows: human TRIM25, NP_005073; mouse TRIM25, NP_033572; zebrafish TRIM25, NP_956469; zebrafish TRIM62, NP_001074143; zebrafish TRIM47, XP_002660960; human TRIM29, NP_036233; mouse TRIM29, NP_076144; human TRIM21, NP_003132; mouse TRIM21, NP_001076021; zebrafish TRIM21, XP_691507; human TRIM39, NP_067076; mouse_TRIM39, NP_840065; zebrafish TRIM39, XP_009292249; human TRIM16, NP_006461; mouse TRIM16, NP_444399; zebrafish TRIM16, XP_009297237; medaka TRIM4-like, XP_011472583. (**b**) Heatmap and phylogenetic analysis for immunity-associated proteins (IMAPs). Accession numbers of other IMAPs from NCBI database are as follows: zebrafish IMAP family member 8 like, XP_002664923; zebrafish IMAP family member 8, XP_009294065; human IMAP family member 8, NP_783161; zebrafish IMAP family member 7, XP_009305485; zebrafish IMAP family member 7 like, XP_009301153; human IMAP family member 7, NP_694968; human IMAP family member 5, NP_060854; zebrafish IMAP family member 4 like, XP_001920359; zebrafish IMAP family member 4, XP_009297228; human IMAP family member 4, NP_060796; human IMAP family member 2 , NP_056475.

**Figure S2**

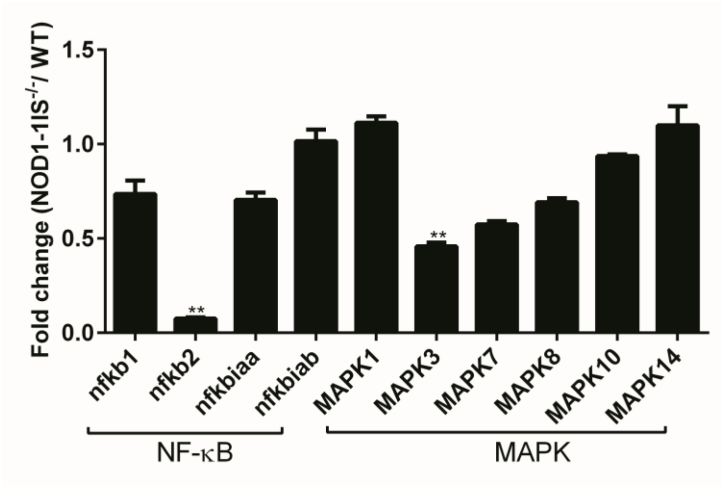


**Fig. S2. The effect of NOD1 deficiency on the expression of those genes involved in NF-κB and MAPK pathways.** 30~50 larvae from WT and *NOD1-1IS*$^{-/-}$ zebrafish were collected at 10 dpf , and used for qRT-PCR. Data represent the mean ± the SEM, and were tested for statistical significance using two-tailed Student's t-test. **$p < 0.01$.

## Figure S3

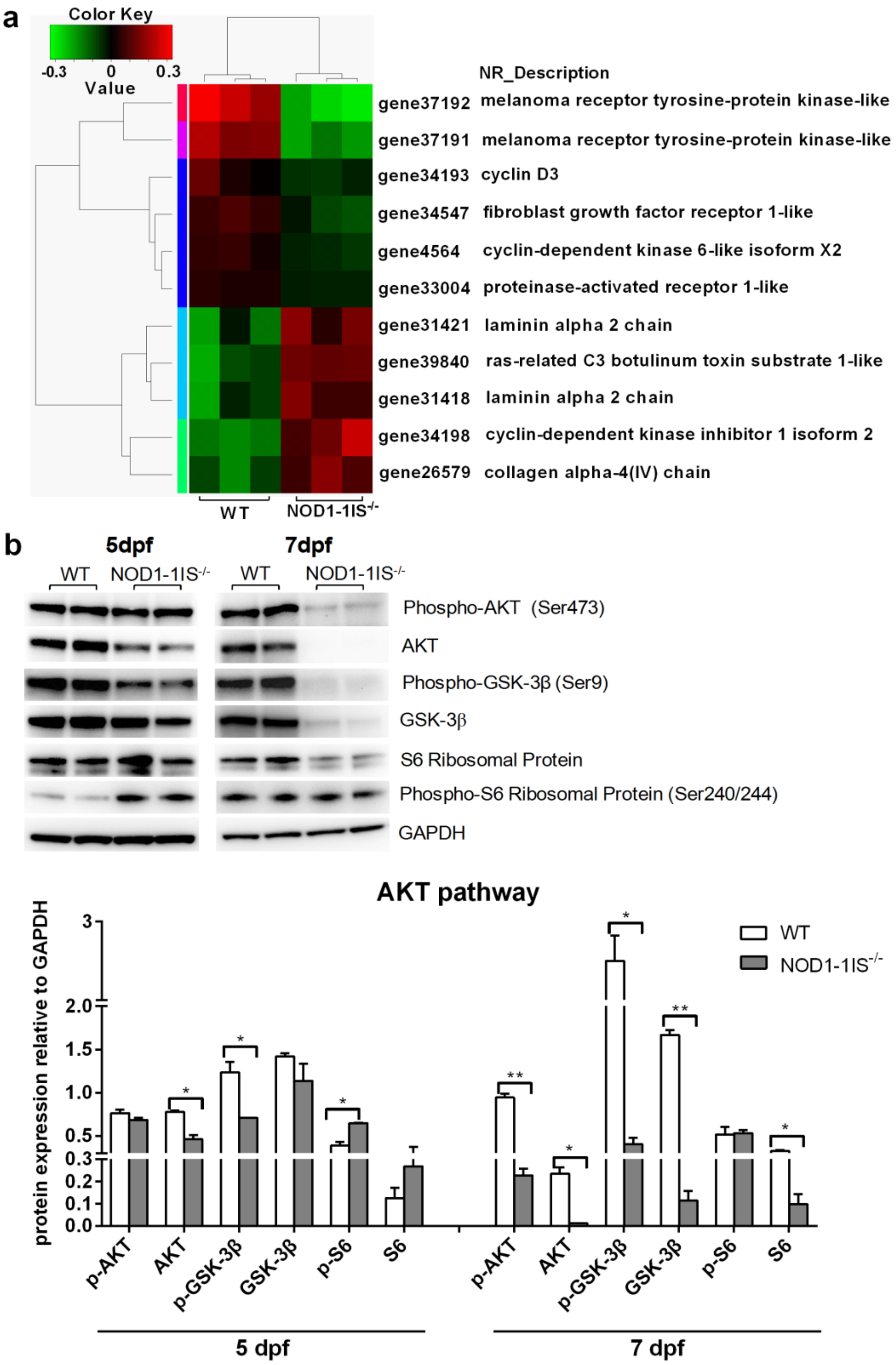


**Fig. S3. PI3K-Akt pathway is impaired in NOD1-deficient zebrafish.** (**a**) Heatmap analysis for differentially expressed genes involved in PI3K/Akt signaling pathway from transcriptomic data. (**b**) Immunoblot analysis of phospho-Akt, Akt, phospho-GSK3β, GSK3β, phospho-S6 and S6 in larvae homogenate from WT and *NOD1-1IS*$^{-/-}$ zebrafish at 5 and 7 dpf. Western blotting results were quantified using Quantity One software. Data represent the average of two independent experiments. *$p$ < 0.05, **$p$ < 0.01.

**Figure S4**

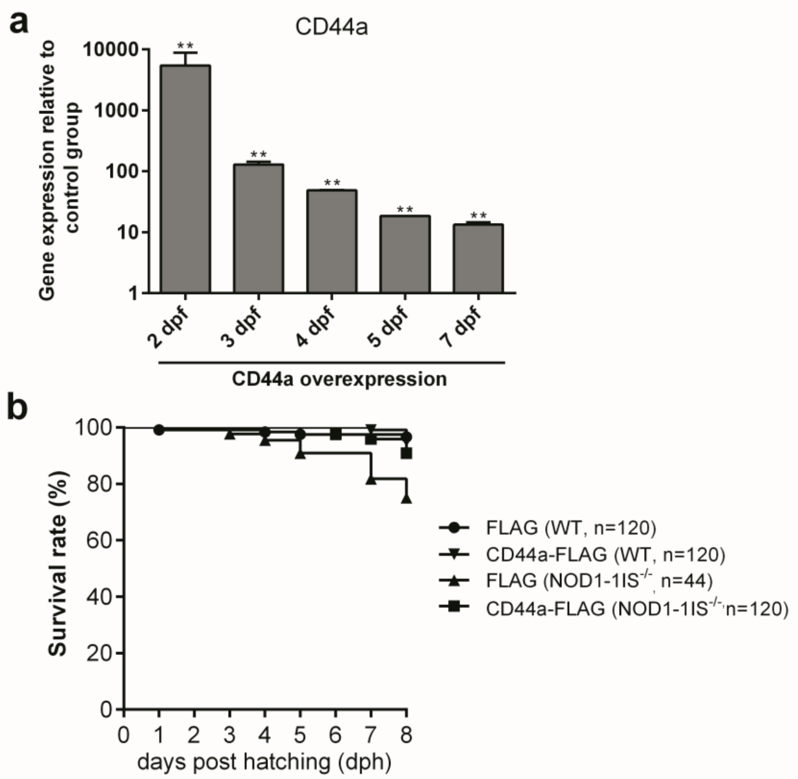


| Log-Rank Test: P values | FLAG (WT) | CD44a-FLAG (WT) | FLAG (NOD1-1IS$^{-/-}$) | CD44a-FLAG (NOD1-1IS$^{-/-}$) |
|---|---|---|---|---|
| FLAG (WT) | X | 0.5403 | **<0.0001** | **0.0683** |
| CD44a-FLAG (WT) | 0.5403 | X | **<0.0001** | **0.1979** |
| FLAG (NOD1-1IS$^{-/-}$) | **<0.0001** | **<0.0001** | X | **0.0056** |
| CD44a-FLAG (NOD1-1IS$^{-/-}$) | 0.0683 | 0.1979 | **0.0056** | X |

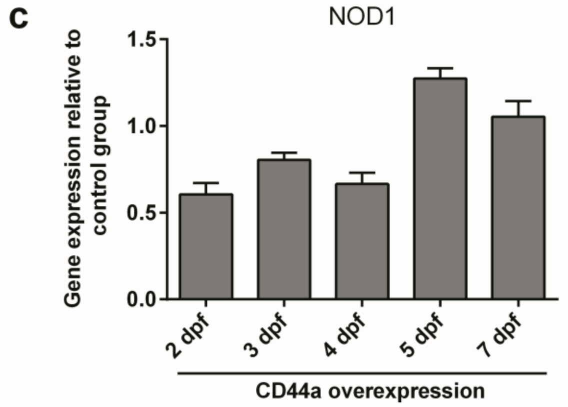


**Fig. S4. CD44a overexpression in *NOD1-1IS*$^{-/-}$ zebrafish rescues larval survival.** (**a**) The expression of CD44a in WT zebrafish microinjected with p3×FLAG or CD44a-FLAG using qRT-PCR (mean ±SEM, n=3). Samples were collected at 2, 3, 4, 5 and 7dpf, and used for RNA extraction. Data were tested for statistical significance using two-tailed Student's t-test. **$p$ < 0.01. (**b**) Survival of WT and *NOD1-1IS*$^{-/-}$ zebrafish microinjected with p3×FLAG or CD44a-FLAG (n=44~120 larvae each group). The survival curves lasting for 8 dph were compared statistically significant difference using the Log-Rank Test. (**c**) The expression of NOD1 in WT zebrafish microinjected with p3×FLAG or CD44a-FLAG using qRT-PCR (mean ±SEM, n=3). Samples were collected at 2, 3, 4, 5 and 7dpf, and used for RNA extraction. Data were tested for statistical significance using two-tailed Student's t-test.

**Table S1. Enrichment analysis of the KEGG pathways for differentially expressed genes in WT vs *NOD1-1IS*$^{-/-}$ zebrafish larvae**

| #Term | Id | Sample number | Background number | P-Value | typeII |
|---|---|---|---|---|---|
| Selenocompound metabolism | ko00450 | 4 | 22 | 0.000649471 | Metabolism of other amino acids |
| Antigen processing and presentation | ko04612 | 10 | 97 | 0.001201018 | Immune system |
| Drug metabolism - cytochrome P450 | ko00982 | 4 | 47 | 0.008010135 | Xenobiotics biodegradation and metabolism |
| Steroid hormone biosynthesis | ko00140 | 4 | 47 | 0.008010135 | Lipid metabolism |
| Metabolism of xenobiotics by cytochrome P450 | ko00980 | 4 | 51 | 0.010417054 | Xenobiotics biodegradation and metabolism |
| Primary bile acid biosynthesis | ko00120 | 3 | 29 | 0.013136656 | Lipid metabolism |
| Retinol metabolism | ko00830 | 4 | 55 | 0.013247944 | Metabolism of cofactors and vitamins |
| NOD-like receptor signaling pathway | ko04621 | 4 | 73 | 0.031766049 | Immune system |
| Carbon fixation pathways in prokaryotes | ko00720 | 2 | 21 | 0.04889759 | Energy metabolism |

**Table S2. Primer information**

| Name | Sequence | Application |
|---|---|---|
| CD44a-FLAGF | GTCAAGCTTACCATGTGGACTTTGTTATTTGTAG | Ligated to p3xFLAG-CMV™-14 vector |
| CD44a-FLAGR | GAAGGTACCATTAAATATTCTTTTTCGTGTTCATC | |
| NOD1-FLAGF | GTGGAATTCTGAAATGAAATTAAATATGG | |
| NOD1-FLAGR | GAAGGATCCGCTGACTCCCTCTCGTTG | |
| NOD2-FLAGF | CCGGAATTCAACTATGAACGCTCAACAGT | |
| NOD2-FLAGR | CGCGGATCCGAAGGTCAATCTGCATTC | |
| NOD1F | AGCAACTGCGACCCTCTTTCA | Quantitative real-time PCR |
| NOD1R | TGGCGAGATATTTCCCACCTACA | |
| CD44aF | GAAAGTAATGCGAAGGAG | |
| CD44aR | TCATCAGTGCCACAATCT | |
| lckF | TACGTAAACATGGGGAACTG | |
| lckR | TCTTCTCCCCTTTCTCAAAC | |
| NALPL1F: | TCGTTCTCCACGGTGACTC | |
| NALPL1R | AGCAGGGCACAAACAATGAC | |
| mhc1umaF | ACGGTGCAAAGGACATACAACAAC | |
| mhc1uma R | ATCAGCCAGTAAATACATCCAAGT | |
| mhc1ulaF | AAGAGTCACATGGGTTTG | |
| mhc1ulaR | CTCATTTGGCTTTAGTTC | |
| similar to mhc1uba-F | GGATGGAAGATAAACTTGGGACA | |
| similar to mhc1uba-R | CAGCCACTCAATGCACTCAGACC | |
| mhc1ubaF | AAAACGCTCACCTGGACTG | |
| mhc1ubaR | ATACTTCTGCACCCACTCA | |
| MHC-IIβF | TGAGGAGATGGCTGATGGAGAC | |
| MHC-IIβR | CATAGATGATGGGCTTAGTTGA | |
| mhc2dabF | CTCTGTGGGGAAGTTTGTG | |
| mhc2dabR | CCAGATCCGAGCATTATGTC | |
| gene26712F | GTGGCATTGATGTGGCAGAAGC | |
| gene26712R | CGACTGACAAACCCAGAAGAAA | |
| gene38616F | AAAACTACTTCGCTCTTCCCTAA | |
| gene38616R | TACAGACTCCTGGATTTCTTGAT | |
| gene26713F | AGGTTGTGCCGCTTTGACTT | |
| gene26713R | CATTCCTTTAGATTGTGGGT | |
| gene2604F | TTTCTTGAGCGGGAATACAGT | |
| gene2604R | ATGAGGGATGGAAGTTTGGAC | |
| gene39147F | GCTCTATTGGCACGATGTTA | |
| gene39147R | CAGACGATTGTTGCGTAGGT | |
| gene4944F | GCATTCAGTGATGGGTCT | |
| gene4944R | ATCCAGGTGTCCAGTTTT | |
| gene12406F | CCTGACACAAGTCGACAAAAGAGAA | |
| gene12406R | ATCCAGATGAGGGAAGAACGAAGC | |
| gene12839F | ACCTCTTTCTGCGTTTCCTGCTT | |
| gene12839R | GCTTCTCCTCCGAGTGTTTGTCT | |
| gene4933F | GAGGACAAAAAAACAACATCAGTC | |
| gene4933R | CTCACAGCGGGCACCAGTCT | |
| gene8198F | TCTTTAGAAGTGGAGGAC | |
| gene8198R | ATGTGGCTGATGATGTTT | |
| gene39994F | TGACTTTGTGGAGGAACT | |
| gene39994R | GCTGAGCGGTGACATTAC | |

| | | |
|---|---|---|
| gene3642F | GATTCCTGATGTCTGGGTC | |
| gene3642R | AGGGTGTAAACATTTCGGT | |
| gene12370F | TCCTGGGCTTTAATCTGCTACG | |
| gene12370R | GCGGCTCAGTGAATGTGGTTTG | |
| gene1131F | CCACGGACCCTCCACAGAACTAT | |
| gene1131R | CAGCGACTCCCTTCGTCAGCACA | |
| gene1132F | AAATCCATCAACCTGTTCCACT | |
| gene1132R | ACAAAGACCACCGCTGACCACT | |
| gene1133F | AGATGAAAACCTGCCCAAA | |
| gene1133R | GCTAGAGCCGTGACAAGTG | |
| gene12961F | TGCTAGAGTTTCTGCTGGAC | |
| gene12961R | AAAGTTTATTGGTGGACGC | |
| gene26693F | CAATGAGAAGTTTGCGGAGTT | |
| gene26693R | ATGACAGCAGATGCGGTTATG | |
| gene33648F | GACCAATCTCATCAGAGGGAA | |
| gene33648R | CGGCGAGTAGCACATAATAAA | |
| gene26589F | AAGTTGGTGGGTTGTGGT | |
| gene26589R | CTCGCAGATTTGAGAAGT | |
| gene6751F | GAGAATACACAGAAGCAAGGTGCAG | |
| gene6751R | GAACGGCAGTGGAAATATGAAGAC | |
| gene23645F | AGAAGCTCGTGCCCGTAGT | |
| gene23645R | ATGCCAAATCAGCAGAACC | |
| nfkb1F | TGTGGTTCGGCTGATGTTC | |
| nfkb1R | GGTTCGCTCGTCTCGTTGT | |
| nfkb2F | AAGATGAGAACGGAGACACGC | |
| nfkb2R | TCTACCAGCAATCGCAAACAA | |
| nfkbiaaF | CATTCACGAGGCAGAGGATTAT | |
| nfkbiaaR | TCCGCTGTAGTTAGGGAAGGTA | |
| nfkbiabF | TGTCATTGTTGGAAAGCCTCAT | |
| nfkbiabR | TAATCCTCGTCGCTATCCTCAC | |
| MAPK1F | ATTACCTGCTGTCCCTTCC | |
| MAPK1R | CTTCCTCCACCTCAATCCTC | |
| MAPK3F | GACCTGAAGCCCTCAAACC | |
| MAPK3R | GCATCCCACAGACCAAATA | |
| MAPK7F | CCTCAGGGAAAGTCACAATG | |
| MAPK7R | TCAGGTATCAGGAAGCGTAG | |
| MAPK8F | TGACCCGTTACTACAGAGCC | |
| MAPK8R | CATCCACCGAGATTCGTTTT | |
| MAPK10F | GGGGCTCAGGGAATAGTGT | |
| MAPK10R | AATCTTCAGCGTGCAGTCG | |
| MAPK14F | CGTGACGGTGGACATTTGGT | |
| MAPK14R | TGGGCATCTGAGGAAGTGAG | |
| GAPDHF | GTAACTCCGCAGAAAAGCCAGAC | |
| GAPDHR | CAAAAGAAACTAACACACACACA | |
